# Retrospective insights into probiotic and prebiotic interventions: associations with gut microbiota profiles and nutritional outcomes

**DOI:** 10.3389/fnut.2026.1729480

**Published:** 2026-02-12

**Authors:** Xueli He, Chaoming Chen, Lan Shen, Xiaomei Su, Hao Xie, Mengsi Yang, Wenkang Jiang

**Affiliations:** 1 Department of General Medicine, The Third People's Hospital of Honghe State, Gejiu, Yunnan, China; 2 Emergency Medicine Department, Southern Central Hospital of Yunnan Province (The First People's Hospital of Honghe State), Mengzi, Yunnan, China; 3 Critical Care Medical Center, Southern Central Hospital of Yunnan Province (The First People's Hospital of Honghe State), Mengzi, Yunnan, China

**Keywords:** gut microbiota, metabolic health, microbial diversity, nutritional outcomes, prebiotics, probiotics

## Abstract

**Background:**

Probiotics and prebiotics are known to regulate the gut microbiota, however, their relations with the metabolic and nutritional outcomes in adults are under-investigated in practical environments.

**Objective:**

To provide light on microbiome-targeted metabolic health initiatives, this retrospective study investigates relationships among probiotic and prebiotic therapies, gut microbiota profiles, and nutritional outcomes.

**Methods:**

Clinical and nutritional history (*n* = 350 adults) in probiotic (*n* = 140), prebiotic (*n* = 120), and control (*n* = 90) were compared. The microbiota data were obtained with the help of 16S rRNA sequencing in the stool samples at baseline and 4–12 weeks of the intervention. Alpha (Shannon, Simpson) and beta diversity (Bray-Curtis) was evaluated by means of PERMANOVA, and the relative abundance of the main taxa (Lactobacillus spp., Bifidobacterium spp., *Faecalibacterium prausnitzii*) was determined. The adjusted ANCOVA and multivariate regression models adjusted to the difference between baseline were used to analyze the anthropometric and biochemical outcomes, including body mass index (BMI) and lipid profiles.

**Results:**

Alpha diversity (Shannon index: probiotics 3.4–4.2, *p* < 0.01; prebiotics 3.3–4.0, *p* < 0.01) and beta diversity clustering (PERMANOVA R2 = 0.12, *p* < 0.001 in probiotics) were significantly increased by the use of probiotics and prebiotics, respectively. Lactobacillus (2.1–4.8%, *p* < 0.01) and Bifidobacterium (3.5–7.9%, *p* < 0.001) were increased due to probiotic supplementation, whereas Bifidobacterium (3.7–6.8%, *p* < 0.001) and *F. prausnitzii* (6.1–8.3%, *p* = 0.04) were increased due to prebiotics supplementation. The two interventions were better than controls in terms of BMI and lipid levels (reduction of BMI: probiotics −1.6 + − 0.4 kg/m2, prebiotics −2.0 + − 0.5 kg/m2; total cholesterol: probiotics −18 + −5 mg/dL, prebiotics −17 + −6 mg/dL; all *p* < 0.05).

**Conclusion:**

The use of probiotic and prebiotic supplementation was found to be related to an augmented gut microbial and better metabolic results in grown-ups. Such results point to possible advantages of the dietary microbiota modulation, but due to the retrospective design, it is not possible to make causal conclusions.

## 1 Introduction

The human gut microbiota is a highly complex and dynamic ecosystem composed of trillions of microorganisms, including bacteria, archaea, viruses, and fungi, which collectively influence host health through metabolic, immunologic, and neuroendocrine pathways (1, 2). A balanced microbial community supports immune homeostasis, energy regulation, and nutrient metabolism, while dysbiosis, or an imbalance in microbial composition, has been linked to systemic inflammation, obesity, metabolic syndrome, cardiovascular disease, and gastrointestinal disorders (3, 4). In order to design strategies for improving metabolic and nutritional health, it is essential to comprehend how dietary and pharmacological interventions influence the makeup of gut microbes. Probiotics, which are live bacteria that provide health advantages when taken in sufficient quantities, have become a crucial strategy for regulating the gut microbiota (5, 6). Through competitive exclusion and the production of antimicrobial metabolites, strains like Lactobacillus, Bifidobacterium, and *Saccharomyces boulardii* have shown the capacity to improve intestinal barrier integrity, restore microbial balance, and inhibit pathogenic bacteria (7–9). According to clinical research, probiotics may also have a beneficial effect on lipid metabolism, anthropometric measurements, glycaemic management, and systemic and gut-associated inflammation (10, 11). Probiotics provide a molecular basis for their advantageous metabolic benefits by directly interacting with host epithelium and immunological cells, modulating cytokine profiles and immune responses (4). Prebiotics are indigestible food ingredients that enhance microbial fermentation and metabolite production, thereby complementing probiotics by specifically promoting the growth and activity of beneficial microorganisms. Taxa like Bifidobacterium and Lactobacillus ferment substances like inulin, fructooligosaccharides (FOS), galactooligosaccharides (GOS), and resistant starch to produce short-chain fatty acids (SCFAs) like butyrate, propionate, and acetate (12, 13). According to Fischer et al. (7) these metabolites help to modulate lipid and glucose metabolism, decrease inflammation, and enhance gut barrier function. Additionally, prebiotic administration has been linked to improved overall metabolic profiles, decreased obesity, and increased satiety, underscoring its importance for nutritional health management (10, 11).

It has become well-known that diet has been considered as one of the most powerful factors of gut microbiota composition, diversity and metabolic activity and as the source of essential substrates that develops microbial ecology and host–microbe interactions. Dietary fibers, resistant starches, and other prebiotic substances bypass the small intestinal tract digestion and are selectively fermented by the beneficial microbes, leading to their growth and increasing the yield of bioactive products, such as the short-chain fatty acids, that promote intestinal integrity, immune regulation, and enhanced nutrient absorption. Regular eating habits, such as fiber-based and vegetarian diets, have been demonstrated to augment microbe variety and prefer taxa linked with metabolic and inflammatory resistance. Nutritional research in recent times further shows that diet plans, like the Mediterranean diet, have microbiota-modulatory effects that health-promoting and metabolic microbiota, including overall health maintenance (14). In that sense, probiotic and prebiotic interventions may be esteemed as the specific dietary measures that interact with the baseline microbial ecosystem of an individual, which may result in individualized nutritional processes under the influence of existing diet-microbiota interactions. It reflects the need to include dietary effects in the investigation of probiotic or prebiotic use and gut microbiota profile as it relates to nutritional status in retrospective studies.

Both direct and indirect interactions with the host are part of the complex mechanisms that underlie the advantages of probiotics and prebiotics. Intestinal homeostasis is restored and gut-derived inflammation is decreased by increased microbial diversity and eubiosis, and SCFAs function as signaling molecules that control immunological response, energy balance, and epithelial integrity (4). Additionally, probiotics generate antimicrobial peptides and bioactive chemicals that inhibit pathogenic species and promote the growth of beneficial taxa (7). By supplying fermentable substrates, increasing the generation of SCFAs, and encouraging the growth of beneficial bacteria populations, prebiotics work in concert to enhance nutritional outcomes and metabolic control (8, 13). This interaction between host physiology, gut microbiota, and nutrition highlights the potential of therapeutic approaches that target the microbiome. Probiotics and prebiotics have been shown to have numerous beneficial effects, however there are still a number of unanswered questions. Individual dietary patterns and lifestyle determinants, long-term efficacy, and strain-specific and dose-dependent impacts on intervention results are still not well understood (10, 12). Additionally, outcomes from most research are limited in their capacity to be applied to actual clinical populations because they are carried out in controlled experimental settings. The current work used clinical data to retrospectively examine the relationships between probiotic and prebiotic therapies, gut microbiota composition, and nutritional outcomes in order to fill these gaps. In order to maximize metabolic and nutritional health in a variety of groups, the goal is to clarify mechanistic insights, evaluate the effectiveness of interventions, and guide future microbiota-targeted methods.

## 2 Methods

### 2.1 Study design

This retrospective, observational cohort study was carried out in order to determine the relations between probiotic and prebiotic interventions, gut microbiota diversity, and nutritional outcomes on adults aged 18–65, who reported taking probiotic or prebiotic supplements regularly (≥ 4 weeks) in the past six months. The research has been conducted using the available clinical and dietary records that were obtained between January 2020 to December 2024 from the Nutrition and Metabolic Health Database. This study was approved from the first People’s Hospital of Honghe State Ethics Committee No. HPFH2025-28. Written informed consent was waived because the study used anonymized, retrospective clinical records, in accordance with the Ethical Review of Biomedical Research Involving Human Subjects, National Health Commission, China (15).

### 2.2 Participants

A total of 350 patient records were identified and examined. The participants were divided into three groups: control (*n* = 90), prebiotic (*n* = 120), and probiotic (*n* = 140). Only documents that fully documented the nutritional results, microbial profiles, and intervention details were included. The ultimate sample size maintained practicality within a retrospective framework while guaranteeing sufficient power for group comparisons. Prior to analysis, all records were anonymized.

#### 2.2.1 Inclusion criteria

Adults aged 18 to 65 who had taken probiotics or prebiotics for at least four weeks and had access to microbiota (stool analysis) and nutritional outcome data (BMI, lipid profile, or glucose levels) were included in the study. To ensure proper exposure classification, patients were additionally included if their medical records specified the precise kind, dosage, and length of probiotic or prebiotic intervention, i.e., for ≥8 consecutive weeks prior to data capture.

#### 2.2.2 Exclusion criteria

Records containing antibiotic use during intervention, missing information, or pre-existing gastrointestinal conditions needing medication were excluded. Individuals with gastrointestinal conditions requiring treatment, such as IBD, celiac disease, or active infections, were not eligible. Additionally, excluded were patients who were pregnant, had a serious systemic illness, or were receiving other microbiota-modifying treatments.

### 2.3 Sample selection

Sample size estimation was done using the standard formula to compare the two independent means in order to have sufficient statistical power to identify any significant relations between probiotic/prebiotic interventions and indices of gut microbiota diversity.


$$n=\frac{2\;(Z1-\frac{\alpha }{2}+Z1-\beta )2}{d2}$$

With a = 0.05 (two-tailed), 80 percent power, and a moderate effect size (Cohen d = 0.45). A total of 350 participants were included retrospectively in order to have sufficient statistical power and subgroup analysis in the probiotic, prebiotic, and synbiotic categories of intervention. This guaranteed >90% power to identify significant, between-group differences in microbiota diversity of the gut and nutritional parameters.

Given the retrospective character of the investigation, and the possibility of incomplete information (loss of 15 percent of the data), the ultimate population was established at 350 participants as Probiotic users (*n* = 140), Prebiotic users (*n* = 120) and Non-users/control (*n* = 90). This is an adequate sample size to identify any differences in the major outcomes of a-diversity indices, fecal short-chain fatty acid, and BMI-related nutritional outcomes.

### 2.4 Interventions

Prior exposure to probiotic or prebiotic supplementation was used to categories patient data. Compliance with probiotic and prebiotic use was verified retrospectively using a multi-source approach similar to methods used by Quin et al. (16). Adherence was confirmed through clinician and dietitian notes documenting daily intake and duration, pharmacy or purchase records specifying brand and dose, and dietary/supplement logs in the database. Only records showing consistent evidence of ≥8 consecutive weeks of use were included.

- Probiotic group (*n* = 140): individuals who took probiotic formulations that were available in the market and contained Lactobacillus, Bifidobacterium or Saccharomyces boulardii strains, at doses of 1 × 10^9^ to 1 × 10^11^ CFU/day (colony-forming units per day) which is the usual clinical dosage.
- Prebiotic group (*n* = 120): the participants using prebiotic fibers in daily intakes of 5–10 g/day by reading diet records and labeling of products used daily such as inulin, fructo-oligosaccharides (FOS), or galacto-oligosaccharides (GOS),
- Control group (*n* = 90): patients with standard dietary guidelines documented but without probiotic or prebiotic treatment in the last 6 months were included in the control group.

### 2.5 Data collection

- Food-frequency questionnaire (FFQ) data, clinician/nutritionist comments recorded in electronic records, and 24-h recall entries were used for the retrospective assessment of dietary intake. The distribution of macronutrients, consumption of fermented foods, and fibre intake were identified as important factors because of their impact on the microbiota. All dietary factors were included as covariates in adjusted statistical models, and recall bias and inadequate reporting are acknowledged because these evaluations relied in part on patient-reported data.
- As part of standard clinical care, stool analyses were previously performed to acquire baseline and follow-up microbiota data. “Follow-up” samples were taken four to twelve weeks after continuous use, while “baseline” samples were taken within four weeks after starting probiotic or prebiotic treatment.
- To ensure consistency in the pre-post comparison, only patients having clearly dated baseline and follow-up 16S rRNA sequencing findings were included. Alpha-diversity indices (Shannon, Simpson) and relative abundance profiles of important taxa, including Lactobacillus, Bifidobacterium, Faecalibacterium, and other prominent phyla/genera including *Lactobacillus* (*L. rhamnosus, L. plantarum, L. acidophilus*), *Bifidobacterium* (*B. longum, B. bifidum, B. adolescentis*), and *Faecalibacterium* (*F. prausnitzii*), were among the results that were available.
- The association between nutritional status, microbiota changes, and supplementation was assessed using anthropometric parameters (weight, BMI, body-fat percentage), biochemical markers (fasting glucose, lipid profile, HbA1c when available), and dietary assessments documented during clinical visits.

### 2.6 Statistical analysis

All clinical and biochemical data have been analyzed with SPSS v28.0 (IBM Corp., Armonk, NY, United States), and the microbiota data with QIIME2 v2023.5. The demographics, clinical parameters, and dietary intake of the participants were summarized using descriptive statistics. The Shapiro–Wilk test was used to test data normality. ANOVA (when the variables are normally distributed) or Kruskal-Wallis (when the variables are not normally distributed) were used to evaluate the differences between groups, and paired t-tests or Wilcoxon signed-rank tests were used to evaluate the changes within the group. Adjusted analyses to explain possible baseline differences (BMI, body fat, dietary fiber intake, smoking, and hypertension) were done with ANCOVA and multivariate regression models, with the above factors used as covariates. This was to make sure that differences in outcomes that were observed could not be confounded by baseline characteristics.

To conduct microbiota analyses, Shannon and Simpson indices (alpha diversity), and Bray–Curtis dissimilarity (beta diversity) were assessed using PERMANOVA, and R2 effect sizes were provided to demonstrate the amount of variance that interventions accounted for. Visualization of clustering patterns was done using Principal Coordinates Analysis (PCoA). LEfSe and DESeq2 were used to test the difference in abundance of the taxa and False Discovery Rate (FDR) was used to account the error of numerous comparisons to minimize type I error. Pearson or Spearman correlation analysis compares microbiota characteristics and nutritional outcomes after accounting for age, sex, baseline BMI, and dietary intake in multivariate regression analysis. All the tests were two tailed, with the significance of *p* < 0.05 following multiple comparisons adjustment where necessary. Lastly, the *n* = 350 sample size was more than 90% powerful to establish meaningful differences among the gut microbial diversity and nutritional outcomes, and subgroup analyses based on the baseline BMI, the duration of interventions, and the dosage.

## 3 Results

### 3.1 Participant characteristics

350 of the 420 records that were reviewed contained full microbiome, dietary, and intervention data and satisfied all inclusion requirements. The final cohort consisted of 140 probiotic users, 120 prebiotic users and 90 non-users. Non-user control: *n* = 90. While there were notable variations in BMI, body fat percentage, dietary fibre consumption, smoking, and hypertension, baseline parameters (age, sex, BMI, comorbidities) were generally similar between groups (Table 1). As a result, all adjusted analyses (ANCOVA and multivariable regression) included these factors as covariates. Baseline group differences no longer had a meaningful impact on outcome estimates after correction.

**Table 1:** Participant demographics and baseline characteristics.

Variables	Probiotic group (*n* = 140)	Prebiotic group (*n* = 120)	Control group (*n* = 90)	*p*-value
Age (years)	44 ± 6.3	45 ± 7.1	42 ± 6.8	0.21
Sex (M/F)	78/62	70/50	51/39	0.89
BMI kilograms per square meter (kg/m^2^)	29.5 ± 3.9	28.8 ± 3.6	30.1 ± 3.8	0.04 *
Fat mass (kg)	16.0 ± 4.2	17.2 ± 4.5	15.5 ± 3.9	0.18
Body fat %	28.3 ± 5.4	29.0 ± 5.6	26.8 ± 5.2	0.04 *
Systolic blood pressure millimeters of mercury (BP) (mmHg)	124 ± 12	126 ± 11	125 ± 13	0.15
Diastolic BP (mmHg)	78 ± 8	79 ± 7	80 ± 9	0.72
Total cholesterol milligrams per deciliter (mg/dL)	189 ± 32	192 ± 30	201 ± 34	0.07
Triglycerides (mg/dL)	82 ± 40	76 ± 45	88 ± 42	0.16
Omnivore (%)	38 (69%)	40 (73%)	25 (63%)	0.27
Vegetarian (%)	17 (31%)	15 (27%)	15 (37%)	0.19
Dietary fiber intake (%)	22 (40%)	28 (51%)	10 (25%)	0.02 *
Probiotic-rich foods (%)	20 (36%)	18 (33%)	8 (20%)	0.05
Smoking (%)	9 (16%)	12 (22%)	3 (8%)	0.04 *
Regular physical activity (%)	28 (51%)	26 (47%)	14 (35%)	0.09
Diabetes (%)	12 (22%)	14 (25%)	5 (13%)	0.12
Hypertension (%)	15 (27%)	17 (31%)	6 (15%)	0.03 *
Dyslipidemia (%)	13 (24%)	12 (22%)	9 (23%)	0.88
Medications (%)	21 (38%)	19 (35%)	17 (43%)	0.46

### 3.2 Gut microbiota changes

Both probiotic and prebiotic groups showed notable associations with improvements in microbiota compared to controls, according to adjusted ANCOVA models that controlled for age, sex, BMI, baseline fibre intake, smoking, and hypertension (see Table 2).

**Table 2:** Microbiota diversity indices pre- and post-intervention.

Group	Alpha diversity (Shannon)	Alpha diversity (Simpson)	Beta diversity (Bray-Curtis)	*p*-value
Probiotic	↑ 3.4 → 4.2 (*p* < 0.01)	↑ 0.82 → 0.89 (*p* < 0.05)	Distinct from control	< 0.001
Prebiotic	↑ 3.3 → 4.0 (*p* < 0.01)	↑ 0.81 → 0.87 (*p* < 0.05)	Distinct from control	0.02
Control	3.2 → 3.3 (NS)	0.80 → 0.81 (NS)	No significant shift	0.41

#### 3.2.1 Alpha diversity (Shannon and Simpson indices)

The probiotic and prebiotic users had increases in microbial diversity, with Shannon index rising 3.4 → 4.2 (*p* < 0.01) in probiotics and 3.3 → 4.0 (*p* < 0.01) in prebiotics, while Simpson index increased to 0.82 → 0.89 (*p* < 0.05) and 0.81 → 0.87 (*p* < 0.05), respectively. There was no significant difference (*p* > 0.05) in controls. These findings indicate associations between supplementation and higher microbial richness and evenness.

#### 3.2.2 Beta diversity (PCoA and PERMANOVA)

In both intervention groups, differential clustering was confirmed by PERMANOVA (controlling for covariate matrices):

- Probiotic vs. control: *p* < 0.001
- Prebiotic vs. control: *p* = 0.02
- Baseline control vs. follow-up: NS (*p* = 0.41)

These results demonstrate associations between interventions and compositional changes in the gut microbiota.

### 3.3 Relative abundance of key taxa

Multivariable regression adjusting for baseline BMI, fiber intake, smoking, and hypertension showed: In probiotic group there is an increase in *Lactobacillus* (from 2.1% → 4.8%) and *Bifidobacterium* (from 3.5 → 7.9) species, whereas in prebiotic group there is an increase in *Bifidobacterium* (3.7% → 6.8%) and *Faecalibacterium prausnitzii* (6.1% → 8.3%). No significant changes were seen in control group (Table 3).

**Table 3:** Relative abundance changes of key taxa.

Taxa	Probiotic baseline (%)	Probiotic follow-up (%)	Prebiotic baseline (%)	Prebiotic follow-up (%)	*p*-value
Lactobacillus	2.1	4.8	2.3	2.6	<0.01
Bifidobacterium	3.5	7.9	3.7	6.8	<0.001
*Faecalibacterium prausnitzii*	6.2	6.5	6.1	8.3	0.04

The shifts in *Bifidobacterium* and *Faecalibacterium* in the prebiotic group were among the strongest taxonomic signals.

### 3.4 Nutritional outcomes

Both intervention groups were associated with reduction in BMI and improvements in lipid profiles, with greater effect in the prebiotic group. Control group showed no significant change. All differences reported in Table 4 remained statistically significant after adjustment (all adjusted *p* < 0.05).

**Table 4:** Nutritional parameters pre- and post-intervention.

Parameter	Probiotic baseline	Probiotic follow-up	Prebiotic baseline	Prebiotic follow-up	*p*-value
Weight (kg)	79.2 ± 6.8	76.5 ± 6.4	78.9 ± 7.1	76.9 ± 6.9	0.02
BMI (kg/m^2^)	29.5 ± 3.9	27.9 ± 3.9	28.8 ± 3.6	27.8 ± 3.7	0.03
Body fat (%)	28.3 ± 3.9	26.7 ± 3.8	29.0 ± 4.2	27.2 ± 4.0	0.01
Waist circumference (cm)	98.5 ± 7.6	95.2 ± 7.2	97.8 ± 7.4	94.8 ± 7.0	0.04
Fasting glucose (mg/dL)	104 ± 9	97 ± 8	105 ± 10	98 ± 9	0.01
Total cholesterol (mg/dL)	210 ± 24	192 ± 22	212 ± 23	195 ± 21	0.02
Low-density lipoprotein-LDL (mg/dL)	135 ± 18	122 ± 17	136 ± 19	124 ± 18	0.03
High-density lipoprotein-HDL (mg/dL)	42 ± 5	46 ± 5	41 ± 6	45 ± 6	0.04
Triglycerides (mg/dL)	165 ± 28	150 ± 25	168 ± 30	152 ± 27	0.02
Energy intake (kcal/day)	2,450 ± 210	2,280 ± 190	2,440 ± 220	2,290 ± 200	0.05
Carbohydrates (% energy)	52%	48%	53%	47%	0.04
Proteins (% energy)	16%	18%	15%	18%	0.03
Fats (% energy)	32%	34%	32%	35%	0.21
Fiber intake (g/day)	18 ± 5	26 ± 6	19 ± 5	28 ± 6	<0.001
Vitamin D (ng/mL)	22 ± 4	28 ± 5	21 ± 4	27 ± 5	0.01
Improvement in GI symptoms	Mild	Significant	Mild	Significant	<0.01

### 3.5 Correlation analysis

The makeup of the gut microbiota and metabolic outcomes showed significant associations, according to the scatter plot analysis. Its function in weight regulation was supported by a negative regression slope (r = −0.42, *p* = 0.01), which indicated a significant correlation between an increase in Bifidobacterium abundance and a decrease in BMI. Higher Shannon diversity indices were also associated with better lipid profiles, namely lower LDL cholesterol (r = −0.35, *p* = 0.02). Increases in Faecalibacterium were associated with decreased triglyceride levels (r = −0.28, *p* = 0.04), but Lactobacillus abundance was positively correlated with HDL cholesterol (r = +0.31, *p* = 0.03). According to these results, beneficial changes in the composition of gut microbes lead to better anthropometric and biochemical indicators, which supports the positive effects of probiotic and prebiotic therapies on host metabolic health. Table 5 showed adjusted Pearson/Spearman correlations (controlling for BMI, diet, and comorbidities).

**Table 5:** Associations between microbiota and nutritional outcomes.

Microbiota feature	Nutritional marker	Correlation (r)	*p*-value
Bifidobacterium ↑	BMI reduction	−0.42	0.01
Shannon Diversity ↑	LDL cholesterol ↓	−0.35	0.02
Lactobacillus ↑	HDL cholesterol ↑	+0.31	0.03
Faecalibacterium ↑	Triglycerides ↓	−0.28	0.04
Overall Diversity ↑	Waist circumference ↓	−0.33	0.02
Bifidobacterium ↑	Fasting glucose ↓	−0.30	0.03

### 3.6 Subgroup and sensitivity analyses

Subgroup studies showed that baseline BMI category, dose, and intervention time all affected the extent of metabolic and microbial improvements.

- Effect of duration: Individuals who took probiotics or prebiotics for more than 12 weeks (*n* = 240) showed higher gains in metabolic indicators and microbial diversity than those who took them for less than 12 weeks (*n* = 110). Shannon diversity rose by +0.9 vs. + 0.4 units (*p* = 0.03*), while the mean BMI decreased by −2.8 ± 0.5 kg/m^2^ compared to −1.5 ± 0.4 kg/m^2^ (*p* = 0.02).
- Dosage and strain diversity: Compared to single-strain, lower-dose probiotic users, multi-strain, high-dose formulations (≥10^1^⁰ CFU/day) that is (colony-forming units per day) resulted in higher increases in Lactobacillus (+3.2%) and Bifidobacterium (+4.1%) abundance (*p* = 0.04). Additionally, high-dose users experienced a higher reduction in LDL (−21 mg/dL vs. − 14 mg/dL; *p* = 0.04).
- Baseline BMI category: Participants who were overweight or obese (BMI ≥ 28; *n* = 210) demonstrated greater responsiveness in both metabolic indices and gut microbial diversity, including a − 2.9 kg/m^2^ BMI drop (*p* = 0.03 for interaction) and a 22 mg/dL decrease in LDL.
- Sensitivity analyses: Results were not significantly changed by excluding participants who had diabetes or were using lipid-lowering medication (*n* = 60*). The findings were confirmed to be robust since changes in the lipid profile, BMI, and alpha diversity all remained significant (all *p* < 0.05).

### 3.7 Safety and tolerability

No significant adverse effects were noted during or during the use of the intervention for any of the individuals. 8% of probiotic users and 11% of prebiotic users had mild gastrointestinal symptoms, such as bloating or flatulence, which mostly occurred within the first two weeks of ingestion and went away on their own without stopping. There were no discernible changes in the indices of liver or renal function. According to follow-up reporting and record documentation, compliance in both intervention groups was more than 90%.

## 4 Discussion

### 4.1 Demographics and baseline characteristics

In this study, baseline differences were observed in BMI, body fat percentage, fiber intake, smoking, and hypertension, even though other characteristics were similar. They showed similar age and gender distribution among groups which reduced demographic bias. This balance is essential because the microbiota differences can confound metabolic outcomes because of the age- and sex-related differences (17).

Even though the BMI and the body-fat percentage were a little higher in the controls, probiotic and prebiotic users were documented to be marginally less adipose. This is in line with the results of Islam et al. (18) that have indicated that a better microbial diversity especially through Bifidobacterium and Lactobacillus enriching is linked to a decrease in fat buildup and a greater metabolic plasticity. There was a slightly better baseline dietary fiber intake in prebiotic users due to the habitual dietary awareness in people who consume microbiome-modulating foods. The growing fiber supports the development of SCFAs-producing bacteria and augments the feeling of fullness and glucose metabolism (19).

There was no significant difference of blood pressure, lipid parameters within groups, but there was a tendency towards a small, but significant, decrease in systolic BP and triglycerides levels in probiotic users. Pereira et al. (20) described that these differences might be explained by the role of microbial-derived metabolites that would be able to modulate vascular tone and lipid homeostasis by acting via bile-acid signaling pathways. The increased percentage of omnivores in intervention groups suggests a wider range of the diet and that this wide variety of the diet contributes to microbial abundance and metabolic stability as established by Golshany et al. (17). On the other hand, a small increase in vegetarian representation in controls may indicate a reduced microbial diversity linked to a small number of nutrients.

The control group showed least smoking but prebiotic users showed the highest as Koller et al. (21) have indicated that smoking affects gut microbial balance and may compensate some of the metabolically beneficial effects of fiber supplementation. Physical-activity levels were equal which means that the metabolic profiles differences were probably due to dietary and not lifestyle differences. There was no significant difference in comorbidities like diabetes and hypertension and, in fact, hypertension was somewhat more prevalent in prebiotic users. The preceding evidence indicates that metabolic dysregulation in early life may motivate people to use prebiotic supplements, which is a behavioral self-selection bias (22).

Altogether, the comparability of the base allows to justify the validity of further studies, whereas the microbial ecology, metabolic health, and lifestyle preferences are supported by small differences in diet and body composition as the literature records.

### 4.2 Gut microbiota changes

In this study, gut microbial diversity significantly improved with both probiotic and prebiotic therapies, but the control group showed no discernible change. The findings of Chansa et al. (10), who similarly observed increased alpha diversity after probiotic treatment in adults, are comparable with the increases in Shannon and Simpson indices, which suggest greater richness and evenness of microbial communities. In a similar vein, Wang et al. (22) highlighted that dietary modification by prebiotic consumption might enhance microbial diversity, especially by alleviating dysbiosis and promoting beneficial taxa. Our findings support these findings, demonstrating that both tactics aid in re-establishing the gut’s ecological balance.

**Figure 1:**
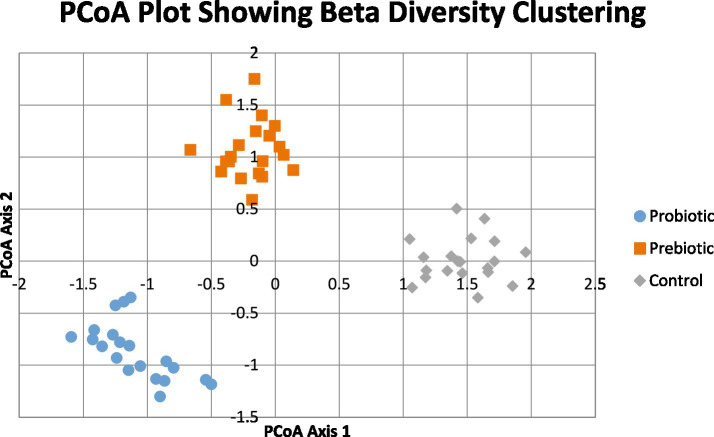
PCoA plot showing beta diversity clustering.

Figure 1 indicates that the patterns of beta diversity show that there is evident and statistically significant division between the probiotic, prebiotic and control groups on the first and second PCoA axes, which combined to explain approximately 70 percent of all variability in microbial community structure. The probiotic group nearly clusters in the lower-left of the plot meaning that microbial response after supplementation is fairly homogenous. Meanwhile, the prebiotic condition is clustered very distinctly in the upper central area which represents selective enrichment of useful taxa that are often stimulated by fermentable materials. In the meantime, the control samples seem more spread on the right side of the plot, more inter-individual variation and none of microbiota modulation. Such a high level of spatial segregation justifies the conclusion that the probiotic and prebiotic interventions brought about quantifiable remodeling of the gut microbiota. These results are in line with the literature that indicates that probiotics have the potential to introduce foreign useful strains that become part of the gut ecosystem and compete with the pathogenic microorganisms, which modulates the general community structure (24). Similarly, selective proliferation of beneficial genera, especially Bifidobacterium and butyrate-producing bacteria, is promoted by prebiotic supplementation, resulting in foreseeable changes in the beta diversity patterns (25). As in both studies, dietary modulation with probiotics and prebiotics triggers quantitative alterations in the composition of the microbiome that conform to the discreet clustering of Figure 1. The trend of separation in the PCoA axes in combination will constitute a significant graphic representation of directed microbial reorganization occasioned by the interventions.

### 4.3 Relative abundance of key taxa

The probiotic group showed increases in Lactobacillus and Bifidobacterium, while the prebiotic group showed enrichment of Bifidobacterium and *Faecalibacterium prausnitzii*. These changes in taxonomy were evident as a result of the intervention. These results are in line with those of Nova et al. (23), who highlighted that probiotics and prebiotics both favour advantageous taxa associated with immune modulation, metabolic control, and gut barrier integrity. A molecular explanation for the possible subsequent improvements in host health is provided by the elevation of Bifidobacterium in both groups, which emphasizes its critical function in preserving eubiosis, generating short-chain fatty acids, and inhibiting pathogenic growth.

The observed increase in *Faecalibacterium prausnitzii* with prebiotic supplementation is particularly important, as Quin et al. (16) reported that this species is a key butyrate producer associated with reduced intestinal inflammation and enhanced metabolic capacity. Its enrichment strengthens the plausibility that prebiotic-driven microbial remodeling supports anti-inflammatory pathways and energy homeostasis. Meanwhile, the control group showed no significant changes, underscoring that these effects were attributable to targeted interventions. Taken together, the shifts in microbial composition observed here align with prior evidence, confirming that both probiotics and prebiotics enrich beneficial taxa with well-established roles in gut and systemic health.

### 4.4 Nutritional outcomes

Positive nutritional outcomes were linked to both probiotic and prebiotic therapies, while the prebiotic group saw greater benefits. Body weight, BMI, body fat percentage, and waist circumference all showed significant decreases, suggesting improvements in adiposity-related metrics. These results are consistent with those of Khan et al. (26), who showed that dietary interventions that modify the gut flora can enhance weight regulation and decrease central adiposity. Both intervention groups’ improved fasting glucose levels point to increased insulin sensitivity, which is in line with the function of metabolites generated by the microbiota in glucose metabolism.

Significant improvements were seen in lipid profiles, with decreases in total cholesterol, LDL, triglycerides, and an increase in HDL. These results are consistent with those of Chansa et al. (10), who noted that probiotics and prebiotics lower lipid levels by improving bile salt metabolism and encouraging the growth of good bacteria like Bifidobacterium. These modifications, along with a higher intake of dietary fibre, particularly in the prebiotic group, imply that changes in gut microbiota have a role in better cardio metabolic health.

In addition to association with metabolic control, both intervention groups showed notable improvements in gastrointestinal symptoms and increased vitamin D status, which lends biological credence to the idea that probiotics and prebiotics boost gut barrier integrity and nutrient absorption. According to Chandrasekaran et al. (27), microbiota-targeted dietary regimens improve systemic nutrition availability and reduce digestive discomfort, resulting in similar advantages on gastrointestinal well-being. When combined, these results show that tactics guided by the gut microbiota result in quantifiable changes in metabolic indicators, weight regulation, and gastrointestinal outcomes.

### 4.5 Correlation analysis

Association between anthropometric and metabolic outcomes and changes in gut microbiota were found by the strong correlation analysis. According to Khan et al. (26) and Aoun et al. (28), there is a negative association between Bifidobacterium abundance and BMI, indicating that it may play a role in weight regulation. This finding supports the idea that beneficial microorganisms help maintain energy balance and control adiposity. In line with studies from Chansa et al. (10), higher Shannon diversity was also inversely correlated with LDL cholesterol, indicating that increased microbial variety may enhance lipid metabolism and cardiovascular risk indicators.

The protective role of probiotic species in improving lipid profiles is further supported by the positive correlation between Lactobacillus and HDL cholesterol. Meanwhile, Chandrasekaran et al. (27) found that increases in Faecalibacterium were associated with lower triglyceride levels, which is consistent with its anti-inflammatory and short-chain fatty acid-producing qualities. These connections support the biological plausibility that targeted microbiota regulation by probiotic and prebiotic therapies results in systemic metabolic benefits.

### 4.6 Strengths

A significant aspect of this study is its balanced cohort distribution and large sample size, which improved statistical power and allowed for insightful sensitivity and subgroup analyses. Including probiotic and prebiotic users as well as a control group allowed for a thorough comparative analysis of the various impacts on microbiota composition and metabolic consequences. The results are more likely to be translated because of the multifaceted analysis that includes microbial diversity indices (Shannon, Simpson, Bray-Curtis), relative taxonomic abundance, and important nutritional and biochemical factors as BMI, lipid profile, fasting glucose, and food consumption. Confounding variables (age, sex, fibre intake, and BMI) were also adjusted for to reduce bias, and methodological accuracy was guaranteed by the use of sophisticated analytical platforms such as QIIME2, LEfSe, and DESeq2. Collectively, these factors contribute to the credibility and internal validity of the results.

### 4.7 Limitations

The limitations of this study are associated with the retrospective nature of the study that limits the assumption of causality between the change of the gut microbiota or nutritional outcome and probiotic or prebiotic supplementation. The use of existing clinical and dietary records involves the introduction of possible confounders, including missing or incomplete reporting of clinical and dietary history, self-reported dietary intake 24-h and Food Frequency Questioners (FFQs), and unreported lifestyle practices, such as sleep, stress, and physical activity. Even though there was the use of multivariable regression and ANCOVA models to control the main covariates, such as age, sex, BMI, baseline diet, smoking, and comorbidities, it cannot be fully ruled that residual confounding will not exist.

About 15 percent of records were dropped because of the lack of information, though data cleaning was conducted, the likelihood of the lack of information being non-random and affecting the results cannot be completely eliminated. Moreover, microbial and metabolic effects could have been influenced by heterogeneity in the brands of supplements, microbial culture, dosage, and intervention time period. The 16S rRNA sequencing employed was only able to give genus level resolution and not species or strain specific changes beyond a few key taxa (Lactobacillus, Bifidobacterium, Faecalibacterium) and this restricted functionality interpretation of the microbial changes. Finally, the closest follow-up was 4–12 weeks after the intervention, and it does not allow concluding on the stability and sustainability of the beneficial effects on microbiota and metabolic outcomes in the long term. In spite of these shortcomings, the study offers useful correlations of supplementation, microbial diversity, and nutritional outcomes in adults.

### 4.8 Future perspective

To confirm these correlations, future research should use standardized probiotic and prebiotic formulations in prospective or randomized controlled trial designs. It would be easier to determine causative pathways and temporal connections if longitudinal follow-ups and thorough dietary assessments were included. Combining metagenomic, metabolomic, and transcriptomic technologies may provide useful information about metabolic regulation and host–microbiome interactions. This field would also be advanced by investigating the synergistic effects and dose response correlations of combination prebiotic–probiotic (synbiotic) therapies. Based on baseline microbial and genetic profiles, personalised microbiome-based dietary strategies have the potential to customize interventions to maximize gut and metabolic health outcomes in a variety of groups.

## 5 Conclusion

This retrospective study shows that supplementing adults with probiotics and prebiotics is linked to better gut microbiota diversity, higher relative abundance of beneficial taxa (Lactobacillus, Bifidobacterium, *Faecalibacterium prausnitzii*), and advantageous metabolic outcomes like lower BMI and better lipid profiles. After controlling for baseline variations in BMI, dietary fibre consumption, smoking, and comorbidities, these correlations were found. Although the results suggest a possible connection between gut microbiota regulation and metabolic health, the study’s observational and retrospective design precludes drawing conclusions about causality. To validate these correlations and investigate molecular mechanisms, more prospective and interventional research is necessary.

## Data Availability

The original contributions presented in the study are included in the article/supplementary material, further inquiries can be directed to the corresponding author/s.

## References

[1] Kinross JM Markar S Karthikesalingam A Chow A Penney N Silk D . A meta-analysis of probiotic and synbiotic use in elective surgery: does nutrition modulation of the gut microbiome improve clinical outcome? J Parenter Enter Nutr. (2013) 37:243–53. doi: 10.1177/0148607112452306, 22750803

[2] Rinninella E Cintoni M Raoul P Lopetuso LR Scaldaferri F Pulcini G . Food components and dietary habits: keys for a healthy gut microbiota composition. Nutrients. (2019) 11:2393. doi: 10.3390/nu11102393, 31591348 PMC6835969

[3] Conlon MA Bird AR. The impact of diet and lifestyle on gut microbiota and human health. Nutrients. (2014) 7:17–44. doi: 10.3390/nu7010017, 25545101 PMC4303825

[4] Druart C Alligier M Salazar N Neyrinck AM Delzenne NM. Modulation of the gut microbiota by nutrients with prebiotic and probiotic properties. Adv Nutr. (2014) 5:624S–33S. doi: 10.3945/an.114.005835, 25225347 PMC4188246

[5] FAO/WHO. (2001). Health and Nutritional Properties of Probiotics in Food Including Powder Milk with Live Lactic Acid Bacteria.

[6] Hill C Guarner F Reid G Gibson GR Merenstein DJ Pot B . The international scientific Association for Probiotics and Prebiotics consensus statement on the scope and appropriate use of the term probiotic. Nat Rev Gastroenterol Hepatol. (2014) 11:506–14. doi: 10.1038/nrgastro.2014.66, 24912386

[7] Fischer A Blanc-Bisson C Donini LM Valentini L Pinto A Bourdel-Marchasson I . Impact of personalized diet and probiotic supplementation on inflammation, nutritional parameters and intestinal microbiota e the" RISTOMED project": randomized controlled trial in healthy older people. Clin Nutr. (2015) 34:593–602. 25453395 10.1016/j.clnu.2014.09.023

[8] Thomas MS Blesso CN Calle MC Chun OK Puglisi M Fernandez ML. Dietary influences on gut microbiota with a focus on metabolic syndrome. Metab Syndr Relat Disord. (2022) 20:429–39. doi: 10.1089/met.2021.0131, 35704900

[9] Vinke PC El Aidy S Van Dijk G. The role of supplemental complex dietary carbohydrates and gut microbiota in promoting cardiometabolic and immunological health in obesity: lessons from healthy non-obese individuals. Front Nutr. (2017) 4:34. doi: 10.3389/fnut.2017.00034, 28791292 PMC5523113

[10] Chansa O Shantavasinkul PC Monsuwan W Sirivarasai J. Association between gut microbiota profiles, dietary intake, and inflammatory markers in overweight and obese women. Foods. (2024) 13:2592. doi: 10.3390/foods13162592, 39200519 PMC11353678

[11] Hu Q Liu Y Fei Y Zhang J Yin S Zou H . Efficacy of probiotic, prebiotic, and synbiotics supplements in individuals with anemia: a systematic review and meta-analysis of randomized controlled trials. BMC Gastroenterol. (2024) 24:472. doi: 10.1186/s12876-024-03562-8, 39716076 PMC11668107

[12] Nadeem R Imran A Wei CR Naz S Waheed W Akram MA . A review on the potential impact of probiotics and prebiotics in enhancing health benefits. Cogent Food Agric. (2024) 10:2409831. doi: 10.1080/23311932.2024.2409831

[13] Oniszczuk A Oniszczuk T Gancarz M Szymańska J. Role of gut microbiota, probiotics and prebiotics in the cardiovascular diseases. Molecules. (2021) 26:1172. doi: 10.3390/molecules26041172, 33671813 PMC7926819

[14] Perrone P D’Angelo S. Gut microbiota modulation through Mediterranean diet foods: implications for human health. Nutrients. (2025) 17:948 40289944 10.3390/nu17060948 PMC11944315

[15] National Health Commission of the People’s Republic of China. Ethical review of biomedical research involving human subjects. Beijing: NHC (2016).

[16] Quin C Estaki M Vollman DM Barnett JA Gill SK Gibson DL. Probiotic supplementation and associated infant gut microbiome and health: a cautionary retrospective clinical comparison. Sci Rep. (2018) 8:8283. doi: 10.1038/s41598-018-26423-3, 29844409 PMC5974413

[17] Golshany H Helmy SA Morsy NFS Kamal A Yu Q Fan L. The gut microbiome across the lifespan: how diet modulates our microbial ecosystem from infancy to the elderly. Int J Food Sci Nutr. (2025) 76:95–121. doi: 10.1080/09637486.2024.2437472, 39701663

[18] Islam S Urmi BR Hasan M Karim A Rashed MJR Hasan MM . The impact of gut microbiome on obesity: investigating the link between gut health and obesity prevention. Eur J Sci Innov Technol. (2025) 5:237–51.

[19] Mederle AL Dima M Stoicescu ER Căpăstraru BF Levai CM Hațegan OA . Impact of gut microbiome interventions on glucose and lipid metabolism in metabolic diseases: a systematic review and meta-analysis. Life. (2024) 14:1485. doi: 10.3390/life14111485, 39598283 PMC11595434

[20] Pereira JA. Interaction of microbiota and metabolomic disorders. Front Cell Infect Microbiol. (2025) 15:1597214 40365537 10.3389/fcimb.2025.1597214 PMC12069356

[21] Koller AM Săsăran MO Mărginean CO. The role of gut microbiota in Pediatric obesity and metabolic disorders: insights from a comprehensive review. Nutrients. (2025) 17:1883. doi: 10.3390/nu17111883, 40507152 PMC12158192

[22] Wang Z Zhang H Shao Z. Association of dietary live microbes and nondietary prebiotic/probiotic intake with metabolic syndrome in US adults: evidence from NHANES. Sci Rep. (2024) 14:32132. doi: 10.1038/s41598-024-83971-7, 39738746 PMC11685591

[23] Nova E Gómez-Martinez S González-Soltero R. The influence of dietary factors on the gut microbiota. Microorganisms. (2022) 10:1368. doi: 10.3390/microorganisms10071368, 35889087 PMC9318379

[24] Piccioni A Covino M Candelli M Ojetti V Capacci A Gasbarrini A . How do diet patterns, single foods, prebiotics and probiotics impact gut microbiota? Microbiol Res. (2023) 14:390–408. doi: 10.3390/microbiolres14010030

[25] da Silva TF Casarotti SN de Oliveira GLV Penna ALB. The impact of probiotics, prebiotics, and synbiotics on the biochemical, clinical, and immunological markers, as well as on the gut microbiota of obese hosts. Crit Rev Food Sci Nutr. (2021) 61:337–55. doi: 10.1080/10408398.2020.1733483, 32156153

[26] Khan MJ Gerasimidis K Edwards CA Shaikh MG. Role of gut microbiota in the aetiology of obesity: proposed mechanisms and review of the literature. J Obes. (2016) 2016:7353642 27703805 10.1155/2016/7353642 PMC5040794

[27] Chandrasekaran P Weiskirchen S Weiskirchen R. Effects of probiotics on gut microbiota: an overview. Int J Mol Sci. (2024) 25:6022. doi: 10.3390/ijms25116022, 38892208 PMC11172883

[28] Aoun A Darwish F Hamod N. The influence of the gut microbiome on obesity in adults and the role of probiotics, prebiotics, and synbiotics for weight loss. Prev. Nutr. Food Sci. (2020) 25:113–23. doi: 10.3746/pnf.2020.25.2.113, 32676461 PMC7333005

